# Clinical Characteristics and Patch Test Results in 57 Patients with Contact Dermatitis in Southern Taiwan

**DOI:** 10.3390/jcm14072291

**Published:** 2025-03-27

**Authors:** Shang-Hung Lin, Yin-Cheng Chao

**Affiliations:** Department of Dermatology, Kaohsiung Chang Gung Memorial Hospital, Chang Gung University College of Medicine, Kaohsiung 833, Taiwan; hong51@cgmh.org.tw

**Keywords:** patch test, contact dermatitis, allergic contact dermatitis, contact allergens, eczematous dermatitis, cobalt

## Abstract

**Background/Objectives**: Patch testing is a valuable clinical tool for identifying the causes of allergic contact dermatitis (ACD). This study aimed to identify common allergens in southern Taiwan. **Methods**: A retrospective review of patch test data from April 2019 to May 2023 was conducted at a tertiary medical center. The European Baseline Series of allergens was utilized to evaluate and identify the causes of dermatitis. The prevalence rates of contact sensitization to each allergen were calculated. **Results**: A total of 57 patients (mean age 41.8 years) with contact dermatitis who underwent patch testing were included. The most common allergens were cobalt chloride (24.6%), followed by fragrance mix I (19.3%), Peru balsam (17.5%), nickel (II) sulfate hexahydrate (15.8%), benzisothiazolinone (15.8%), 4-Phenylenediamine (PPD) base (10.5%), and methyldibromo glutaronitrile (10.5%). Patients with positive patch test results frequently had a history of allergic rhinitis (26.3%), atopic dermatitis (24.6%), urticaria (21.1%), and elevated immunoglobulin E (IgE) levels (28.1%). The hairdressing profession was associated with a higher risk of hand eczematous dermatitis. **Conclusions**: Positive patch test results were observed in 86% of patients diagnosed with contact dermatitis. This study found that cobalt, rather than nickel, was the most prevalent allergen in patients with contact dermatitis. Elevated IgE levels were observed in ACD patients, with the hands being the most frequently affected area. Occupations as accountants, secretaries, and in the hairdressing and cosmetics industries were strongly associated with hand eczematous dermatitis. The early identification of allergens and appropriate treatment strategies significantly reduced recurrence rates and improved outcomes. For individuals with specific allergies, ongoing avoidance of identified allergens is crucial to managing and preventing allergic reactions. Further research is needed to elucidate the mechanisms and responses to novel therapies, including biologic agent- and nanotechnology-based treatments.

## 1. Introduction

Contact dermatitis is a common skin condition caused by repeated exposure to allergens or irritants, leading to the development of allergic contact dermatitis and/or irritant contact dermatitis (ICD) [1].

ACD is relatively common condition, with a prevalence of around 20% in the general population in one large-scale meta-analysis [2]. ACD is also an increasingly prevalent form of dermatitis, resulting from environmental exposure to allergens and mediated by type IV hypersensitivity reactions [3]. The condition begins with a sensitization phase, during which a low-molecular-weight hapten binds to epidermal proteins, triggering an innate immune response and activating skin dendritic cells. These dendritic cells process the hapten–protein complex, migrate to regional lymph nodes, and present the complex to T cells, thereby sensitizing them to the allergen. Sensitized T cells proliferate, forming effector and memory T cells. Upon subsequent exposure to the allergen, the elicitation phase is initiated, resulting in a robust inflammatory response [4,5].

Patch testing is a valuable diagnostic tool used to identify the specific allergens responsible for ACD. The patch test is a simple and safe procedure with no age restrictions. A comprehensive analysis of the patch test results of pediatric groups included infants and young children who were under 3 years old [6]. The broad applications of patch testing extend beyond dermatology, influencing occupational health, consumer safety, and regulatory policies.

The possible applications of patch testing include the identification of allergens responsible for chronic eczematous eruptions and difficult-to-treat dermatitis, screening programs for high-risk professions (e.g., healthcare workers, hairdressers, metalworkers), and the evaluation of cosmetic, pharmaceutical, and personal care products for potential sensitizers. As environmental exposure evolves over time, the allergens that frequently cause reactions may also change. In Taiwan, economic priorities have shifted over the years, moving from labor-intensive manufacturing, to chemical industries, and later to technology industries. Evolving occupational patterns, lifestyles, and consumer habits may have influenced the prevalence of common contact allergens [7].

On the other hand, treatment adherence might be a gradually emerging issue. Poor adherence to treatment was influenced by various factors, including disease chronicity, forgetfulness, treatment inconvenience, therapy misunderstandings, and fear of side effects—particularly regarding topical corticosteroids [8].

This study aimed to identify common allergens in patients from southern Taiwan. The most effective way to prevent ACD was found to be identifying and strictly avoiding possible allergens. For symptom relief, the application of emollients and moisturizers coupled with topical corticosteroid cream and taking antihistamines, systemic corticosteroids, or other immunomodulatory agents might be helpful. To improve treatment outcomes and overall health, novel strategies and good disease-related education that enhance patient confidence must be emphasized. The prevention of work-related contact dermatitis by identifying hazardous substances in occupational settings was important. Therefore, the development of workplace safety guidelines and protective measures should be emphasized if correlations between allergens and ACD are made.

## 2. Materials and Methods

This retrospective cohort review analyzed patients with contact dermatitis who underwent patch testing at the Department of Dermatology, Kaohsiung Chang Gung Memorial Hospital, Taiwan, from April 2019 to May 2023. Data were collected from both outpatient and inpatient medical records, including information on the involved sites, onset time, past history, family history, associated symptoms, comorbidities, trigger factors, and medication use.

Patch testing was conducted using the European Standard Series, following the protocol recommended by the International Contact Dermatitis Research Group (ICDRG). Finn Chambers or IQ Chambers (Epitest Ltd. Oy, Tuusula, Finland) were applied via Scanpor tape (Norgesplaster AS, Vennesla, Norway) to a lesion-free area on the back. To ensure secure adhesion, the chambers were further reinforced with 3M tape (3M, St. Paul, MN, USA). The following substances were all tested allergens: Potassium dichromate, p-Phenylenediamine, Thiuram mix, neomycin sulfate, cobalt chloride, Caine mix 3, nickel sulfate hexahydrate, 2-Hydroxyethyl methacrylate, Colophony, Paraben mix, N-isopropyl-N-phenyl-4-Phenylenediamine, lanolin alcohol, Mercapto mix, Epoxy resin (Bisphenol A), Peru balsam, 4-tert-Butylphenol Formaldehyde resin, 2-Mercaptobenzothiazole, Formaldehyde, fragrance mix I, sesquiterpene lactone mix, Quaternium 15, Propolis, methylchloroisothiazolinone/methylisothiazolinone, Budesonide, Tixocortol-21-pivalate, methyldibromo glutaronitrile, fragrance mix II, Lyral, methylisothiazolinone, and textile dye mix [9].

The patches were removed after 48 h, and the test sites were examined for reactions on day (D) 2 and D4 or, in some cases, on D3 alone. A delayed reading was conducted on D7 for allergens known to cause late positive reactions, as documented in previous studies. These allergens included topical corticosteroids and neomycin for all patients, as well as metals for those with suspected sensitivities. A test result at D3, D4, or D7 was considered positive if the reaction met or exceeded the threshold of palpable erythema, in accordance with ICDRG guidelines. The exclusion criteria were the following situations: patients who were currently under high-dose corticosteroid treatments, those with active and large erythrodermic or eczematous lesions, those who missed scheduled appointments to collect data, and those who lost follow-up after the treatments.

Both the standard and baseline series of allergens were employed to evaluate and identify the causes of contact dermatitis. Reactions were graded using ICDRG criteria: negative (−), doubtful (?+), weak positive (+), strong positive (++), or extreme positive (+++). The prevalence rates of contact sensitization to each allergen were calculated. For patients who exhibited positive reactions to relevant contact allergens, confirming a diagnosis of allergic contact dermatitis, their occupations and the affected dermatitis sites were further analyzed. For mild itching or erythema, lifestyle modifications, topical emollients, topical corticosteroids, and topical calcineurin inhibitors were recommended as the first line of treatment to alleviate symptoms. If the condition worsened or involved a large area, phototherapy and systemic treatments, including oral antihistamines, corticosteroids, or other immunomodulatory agents such as cyclosporine, methotrexate, and azathioprine, were prescribed to manage the disease.

The parameters of possible allergens and occupational relationships were compared with Fisher’s Exact Test. The statistic software used for analysis was IBM SPSS Modeler, version 16.0.

## 3. Results

A total of 57 patients (mean age 41.5 years) with contact dermatitis who underwent patch testing were included in this study (Table 1). There was a female predominance (63.2%), and the mean duration of the symptoms prior to patch testing was 32.6 ± 63.4 months. A total of 15 patients (26.3%) had a past history of atopy (24.6%), 12 patients (21.1%) had a history of urticaria, and 16 patients (28.1%) had elevated IgE levels.

Among the 57 patients, only 8 patients had completely negative findings. A total of 49 patients (86%) were diagnosed with ACD and had at least one positive result for the contact allergens. Of these, 45 patients (91.8%) had clinically relevant reactions. Minimal sensitizations were occasionally noted in few patients, but these were only doubtful or weak positive results that did not cause any discomfort.

The most common allergen was cobalt (24.6%), followed by fragrance mix I (19.3%), Peru balsam (17.5%), nickel (15.8%), benzisothiazolinone (15.8%), 4-Phenylenediamine (PPD) base (10.5%), and methyldibromo glutaronitrile (10.5%).

**Figure 1:**
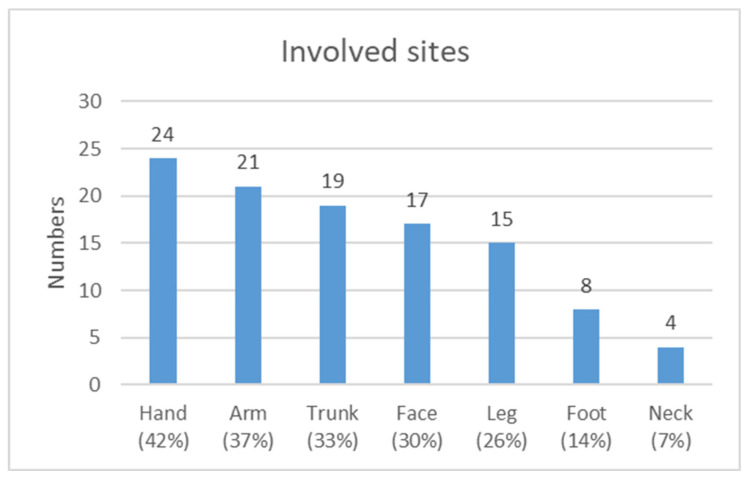
Sites affected by ACD in patients with positive patch test results for contact allergens.

Regarding the sites of involvement (Figure 1), the hands were the most affected area (42%, *n* = 24), followed by the arms (37%, *n* = 21), trunk (33%, *n* = 19), face (30%, *n* = 17), and legs (26%, *n* = 15).

**Figure 2:**
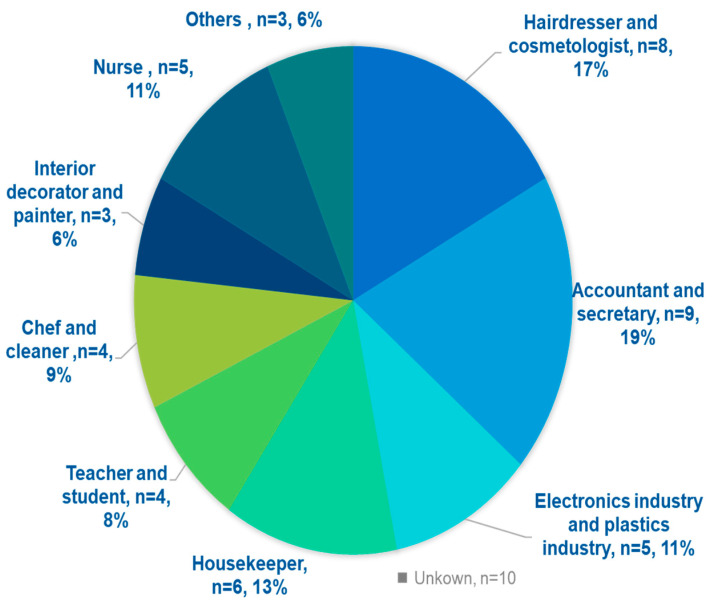
Common occupational distributions associated with ACD.

Figure 2 shows the distribution of common occupations associated with ACD. The most common occupations were accountants and secretaries (19%, *n* = 9), followed by hairdressers and cosmetologists (17%, *n* = 8) and those working in the electronics and plastics industries (11%, *n* = 5). Housekeepers also made up 13% (*n* = 6) of the cohort.

**Figure 3:**
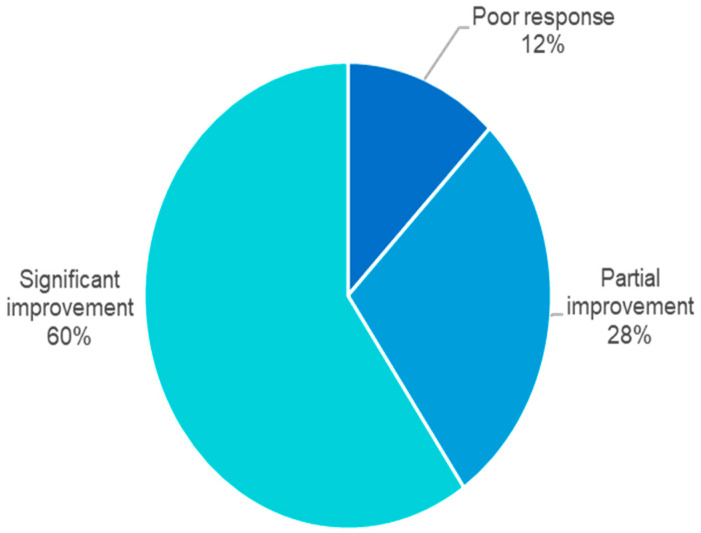
Outcomes following appropriate treatment and avoidance of potential allergens in patients with ACD.

After actively avoiding potential allergens and receiving appropriate treatments, the mean time to improvement was 4.77 weeks. Of the patients, 60% showed significant improvement (erythema and affected area improved more than 80%), 28% showed partial improvement (erythema and affected area improved between 20 and 80%), and 12% had a poor response or recurrence (Figure 3).

**Figure 4:**
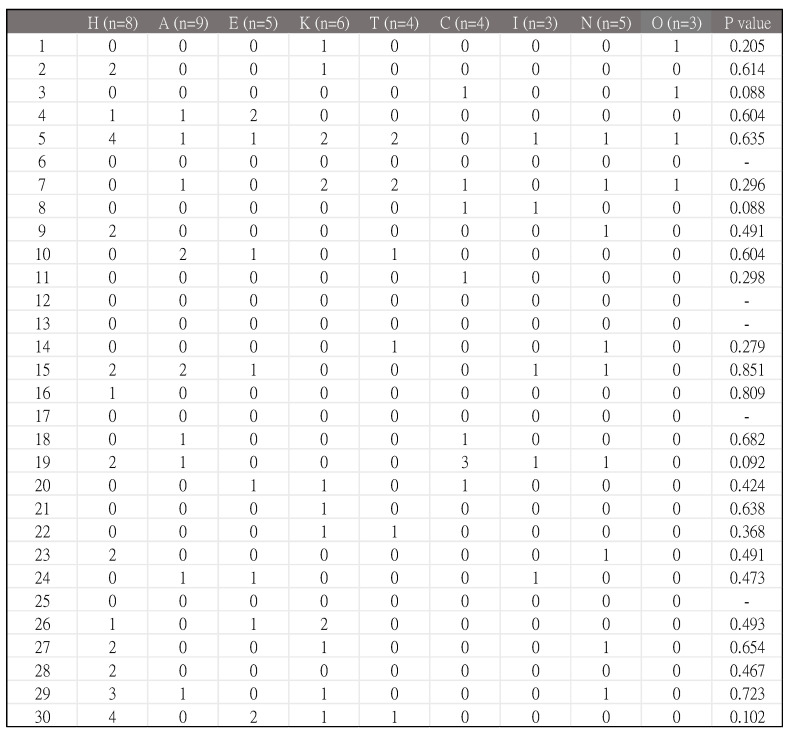
Possible allergens and occupational relationships, compared with Fisher’s Exact Test. Statistic software used: IBM SPSS Modeler, version 16.0. Subgroups: H (hairdressers and cosmetologists); A (accountants and secretaries); E (electronics industry and plastics industry workers); K (housekeepers); T (teachers and students); C (chefs and cleaners); I (interior decorators and painters); N (nurses); O (others).

In addition, possible allergens and occupational relationships were compared with Fisher’s Exact Test (Figure 4). However, all *p* values were >0.05, meaning that there were no strong relationships between allergens and occupations in our study.

## 4. Discussion

Our study revealed that cobalt was the most prevalent allergen among patients with ACD. Furthermore, an increase in cases of hand ACD was noted, likely linked to frequent occupational exposure to allergens, especially among accountants and secretaries and hairdressers and cosmetologists. Patients with positive patch test results often had other skin conditions and elevated IgE levels. The average duration of the disease was 32.63 months, with a mean improvement time of 4.77 weeks, highlighting that the early identification of allergens and appropriate treatments were key factors in recovery.

Dermatitis may recur upon re-exposure to allergens. A negative result from patch testing should not immediately rule out the possibility of ACD. Clinicians often consider various factors, such as the formulation and concentrations of the chemicals tested, the relevance of the allergen series to the patient’s occupational exposures, potential immune system suppression, or the emergence of new allergens [4]. Additionally, ICD can clinically resemble ACD and may not always produce positive patch test results, despite being associated with occupational exposures to chemicals or physical agents [10]. Notably, data from the European Surveillance System on Contact Allergies has indicated that even in cases ultimately diagnosed as ICD, patch testing can still yield positive results.

The positive rate of patch testing in patients diagnosed with contact dermatitis was 86%. In other studies, nickel has been identified as the most frequently encountered allergen in patients undergoing patch testing globally, with common sources of nickel exposure including buttons, belt buckles, eyeglass frames, and various types of jewelry [7,11,12]. However, cobalt was the most common allergen in our study.

A cobalt allergy can be triggered by various everyday items and materials. Individuals with this sensitivity may experience allergic reactions when exposed to cobalt, which is commonly found in consumer products such as jewelry, metal household items (cutlery, zippers, coins, and keys), cosmetics (eye shadow, blushers, and compact powders), leather goods (accessories, clothing, and shoes), hair dye, medical devices (orthopedic implants, stents, pacemakers, and medication pumps), dental alloys, cement, plastics, ceramics, paints, and printing inks (books, magazines, and packaging). In recent years, the rise in the use of personal electronic devices has introduced new sources of nickel and cobalt sensitization. Mobile phones and laptops have become additional contributors to nickel and cobalt allergies, as these devices often contain components made from these metals.

Previous studies have shown trends in cobalt sensitization that vary regionally [7]. For instance, another study in Taiwan revealed a consistent increase in the prevalence of positive reactions to cobalt [7], while data from Germany showed stable rates from 1992 to 2012 [13]. In contrast, a study in northern Italy observed an initial increase in cobalt allergies from 1996 to 2010, followed by a decline [14]. These trends emphasize the potential impact of regional factors on cobalt sensitivity while highlighting the need for ongoing monitoring and localized studies to help clarify the factors influencing cobalt sensitization in different populations.

Fragrances are among the second or third most common allergen group to cause ACD [11,15]. The popularity of cosmetics in the Western world began in the 17th century. Initially, they were used by women who had recovered from smallpox to cover the scars on their faces. The purpose of using cosmetics was to make oneself more attractive. For most women and men, using cosmetics was a way to appear healthier and younger. As science began to explore the human body more deeply, research into skin aging and deterioration was launched. Thanks to technology, the quality of beauty products has improved rapidly, and people have become increasingly aware of using various skincare products to maintain their skin, hoping to appear both younger and healthier when applying makeup. Cosmetics are products applied to the body or used in similar ways to clean, beautify, enhance charm, alter appearance, and maintain the health and beauty of the skin and hair. In this era, with the popularity of cosmetics and skincare and haircare products, allergies to their ingredients have become increasingly common. In summary, comparing our results to other large studies [7,16,17], cobalt, nickel, and fragrance mix I were among the top five most common allergens (Table 2).

Regarding the affected sites (Figure 2), the hands were the most affected area, followed by the arms, trunk, face, and legs. In a recent study, the hands and face were the most commonly reported anatomical sites of dermatitis, with approximately 45% of cases of ICD being occupational in origin [18].

Occupational hand dermatitis is a prevalent condition worldwide. ICD is more common than ACD, though their prevalences vary by region. According to data from the US Bureau of Labor Statistics, the incidence rate of occupational skin diseases in 2020 was 1.8 cases per 10,000 full-time equivalent workers annually, making it the second most common nonfatal occupational illness [19]. Occupational hand dermatitis can greatly affect an individual’s quality of life [20]. The Centers for Disease Control and Prevention reports that around 90% of occupational skin conditions are classified as contact dermatitis, with up to 80% involving the hands [21]. Hand dermatitis can be challenging to differentiate from other inflammatory processes such as psoriasis or dyshidrotic eczema [22]. Chronic urticaria and protein contact dermatitis are much less frequent [23]. Frequent exposure to water or moisture is a significant risk factor for hand dermatitis [24,25]. Repeated exposure can cause the maceration of the stratum corneum, impair its barrier function, and increase susceptibility to irritants and allergens [26]. In our study, cobalt and methylchloroisothiazolinone/methylisothiazolinone were the most frequently identified allergens in patients with hand eczema, followed by fragrance mix I and nickel. This finding aligns with a retrospective study conducted by the Danish Contact Dermatitis Group, which reported nickel as the most common contact allergen among individuals with hand eczema. Other prevalent allergens in their study included methylchloroisothiazolinone/methylisothiazolinone, cobalt, and fragrance mix I [16].

Possible relationships between allergens and occupations were compared with Fisher’s Exact Test (Figure 4). However, all *p*-values were >0.05, meaning that there were no strong relationships between allergens and occupations in our study. Nevertheless, some patients with ACD still had occupational associations in real-world practice.

Accountants and secretaries were the most commonly affected occupations, with cobalt, Peru balsam, and Formaldehyde being common allergens. These allergens are often found in writing materials, ink, tickets, stamps and seals, note paper, and banknotes, with prolonged exposure leading to hand dermatitis. Contact dermatitis caused by substances used in hair and nail grooming and adornment is one of the most common occupational diseases among cosmetologists [27]. In our study, hairdressers and cosmetologists were the second most common set of occupations associated with contact dermatitis. Cobalt, PPD, fragrance mix I, and methylisothiazolinone were the most common allergens identified in these groups.

Healthcare workers and housekeepers also experience a higher prevalence of occupational contact dermatitis than the general population. Frequent wet work in these professions, such as hand sanitation and glove use, contributes to skin irritation [28,29].

One study found that over 90% of healthcare and cleaning workers reported symptoms of hand dermatitis, underscoring the substantial burden of this condition in these occupations [30]. Interventions and workplace strategies are needed to mitigate the impact of occupational dermatitis.

Gloves, a critical component of personal protective equipment for healthcare and cleaning workers, are a significant source of ACD. A previous study identified rubber additives as a common cause of glove-related ACD [31]. Diagnosing glove-related ACD can be complex, as some additives used to enhance flexibility and durability are frequent sensitizers. Identifying suitable glove brands can reduce allergic reactions and improve safety in medical settings [32]. The prevention of work-related contact dermatitis by identifying hazardous substances in occupational settings is important. Therefore, the development of workplace safety guidelines and protective measures should be emphasized if correlations between allergens and ACD are made.

In our study, patients with positive patch test results tended to have a history of allergic rhinitis (26.3%), atopic dermatitis (24.6%), urticaria (21.1%), and elevated IgE levels (28.1%). Elevated IgE levels were associated with type I hypersensitivity, which arising from exposure to antigens with protein molecular weights typically between 10 and 40 kDa [33]. In contrast, type IV hypersensitivity is driven by T cells triggering an inflammatory response against either exogenous or endogenous antigens [3]. Some reports mentioned that 72 h positive patch test responses were strongly correlated with total serum IgE levels and symptom severity index scores. Some positive patch test reactions were regarded as late phases of type I allergic reactions [34].

Patients with atopic dermatitis exhibit increased allergen penetration and immune dysregulation—including shared cytokine pathways—and the frequent use of emollients and topical medications, all of which may contribute to the development of ACD. Recent systematic reviews suggest that ACD is a significant clinical concern in both children and adults with atopic dermatitis. Although this remains a topic of debate, ACD is recognized as an important comorbidity and a potential trigger for atopic dermatitis exacerbations in clinical practice [35]. An analysis of 1142 pediatric patch test cases from the Pediatric Contact Dermatitis Registry found that 30% were diagnosed with both atopic dermatitis and ACD simultaneously [36]. A 2021 review by the North American Contact Dermatitis Group reported similar findings, with 29.5% of children (*n* = 1648) and 20.7% of adults (*n* = 36,834) diagnosed with both atopic dermatitis and ACD concurrently [37]. Common allergens relevant to atopic dermatitis include lanolin, neomycin, Formaldehyde, sesquiterpene lactone mix, compositae mix, and fragrances, frequently present in personal care products [35].

For mild itching or erythema, lifestyle modifications, topical emollients, topical corticosteroids, and topical calcineurin inhibitors are recommended as the first line of treatment to alleviate symptoms. The prevalence of ACD to topical corticosteroids was very low (around 1.5%), but it is still an issue due to their increasing use in diverse dermatologic conditions. If some lesions are progressing under topical corticosteroids use, patch tests might be considered to clarify suspicions about the cause of treatment failure [38]. Physicians should also be aware of the side effects of topical corticosteroids or tacrolimus, including telangiectasias, skin atrophy, and rosacea-like granulomatous dermatitis. Early diagnosis and timely discontinuation are crucial if some signs are noted during treatments [39,40].

If the condition worsened or involved a large area, phototherapy and systemic treatments, including oral antihistamines, corticosteroids, or other immunomodulatory agents such as cyclosporine, methotrexate, and azathioprine, were prescribed to manage the disease. Eosinophilia also decreased with appropriate treatments and after removing the allergens.

After suitable treatments, most patients experienced high levels of disease improvement, with 60% achieving significant recovery (erythema and affected area improved more than 80%) and 28% achieving partial improvement (erythema and affected area improved between 20 and 80%). However, 12% of patients still had poor responses or recurrences, necessitating additional treatments.

For promising novel therapies, biologic agents like dupilumab have shown promising effects, while others such as ustekinumab have only shown limited efficacy [12]. There is some potential for Withaferin A (a phytochemical compound) in treating complicated dermatological diseases [41]. Additionally, topical nanotechnology-based therapies also demonstrate fabulous responses due to their deeper drug permeation, targeting characteristics, and ability to reduce adverse effects [42]. Further research is still needed to elucidate the mechanisms of and responses to novel therapies, including biologic and nanotechnology-based treatments.

Our study had several limitations, including a small sample size from a single center, a retrospective design with some unstable data qualities, and potential selection bias. Large-scale epidemiological studies and further prospective studies to assess the impact of occupational exposure on contact dermatitis are needed to provide more robust data.

## 5. Conclusions

This study found that cobalt, rather than nickel, was the most prevalent allergen in our patients with contact dermatitis. Elevated IgE levels were observed in ACD patients, with the hands being the most frequently affected area. Occupations as accountants, secretaries, hairdressers, and those in the cosmetics industry were strongly associated with hand eczematous dermatitis. The early identification of allergens and appropriate treatment strategies significantly reduced recurrence rates and improved patient outcomes. For individuals with specific allergies, the ongoing avoidance of identified allergens is crucial to managing and preventing allergic reactions. Further research is needed to elucidate the mechanisms and responses to novel therapies, including biologic agent- and nanotechnology-based treatments.

## Figures and Tables

**Table 1:** Baseline characteristics of patients with patch testing for ACD.

Demographic Data	Total Patients
Age (years), mean (SD)	41.5 (15.2)
Sex, Female, *n* (%)	36 (63.2)
Duration (months), mean (SD)	32.6 (63.4)
Allergic rhinitis history, *n* (%)	15 (26.3)
Atopy history, *n* (%)	14 (24.6)
Urticaria history, *n* (%)	12 (21.1)
Elevated IgE levels, *n* (%)	16 (28.1)
Positive for at least one allergen, *n* (%)	49 (86.0)
Clinical relevance, *n* (%)	45 (91.8)

**Table 2:** Cobalt, nickel, and fragrance mix I were among the top five most common allergens.

Our Study (Southern Taiwan)	Positive Reactions, *n* = 57 (%)	Lin et al. [7] (Taiwan)	Positive Reactions, *n* = 4005, (%)	Boonstra MB et al. [16] (Denmark)	Positive Reactions, *n* = 1571, (%)	Sukakul T et al. [17] (Thailand)	Positive Reactions, *n* = 2803, (%)
Cobalt	24.6	Nickel	18.2	Nickel	18.8	Nickel	24
Fragrance mix I	19.3	Cobalt	7.7	MCI/MI	9.2	Methylisothiazolinone	14.6
Peru balsam	17.5	Medicaments	7.6	Cobalt	6.8	Potassium dichromate	13.1
Nickel	15.8	Fragrance mix I	6.7	Fragrance mix I	6.2	Fragrance mix I	12.6
Benzisothiazolinone	15.8	Chromium	4.9	MDBGN	6	Cobalt	10.5

MCI/MI, methylchloroisothiazolinone/methylisothiazolinone; MDBGN, methyldibromo glutaronitrile.

## Data Availability

Data were contained within the article.
